# Informal Urban Settlements and Cholera Risk in Dar es Salaam, Tanzania

**DOI:** 10.1371/journal.pntd.0000631

**Published:** 2010-03-16

**Authors:** Katherine Penrose, Marcia Caldas de Castro, Japhet Werema, Edward T. Ryan

**Affiliations:** 1 Department of Global Health and Population, Harvard School of Public Health, Boston, Massachusetts, United States of America; 2 Ministry of Lands and Human Settlements Development, Dar es Salaam, Tanzania; 3 Division of Infectious Diseases, Massachusetts General Hospital, Boston, Massachusetts, United States of America; 4 Department of Medicine, Harvard Medical School, Boston, Massachusetts, United States of America; 5 Department of Immunology and Infectious Diseases, Harvard School of Public Health, Boston, Massachusetts, United States of America; Weill Medical College of Cornell University, United States of America

## Abstract

**Background:**

As a result of poor economic opportunities and an increasing shortage of affordable housing, much of the spatial growth in many of the world's fastest-growing cities is a result of the expansion of informal settlements where residents live without security of tenure and with limited access to basic infrastructure. Although inadequate water and sanitation facilities, crowding and other poor living conditions can have a significant impact on the spread of infectious diseases, analyses relating these diseases to ongoing global urbanization, especially at the neighborhood and household level in informal settlements, have been infrequent. To begin to address this deficiency, we analyzed urban environmental data and the burden of cholera in Dar es Salaam, Tanzania.

**Methodology/Principal Findings:**

Cholera incidence was examined in relation to the percentage of a ward's residents who were informal, the percentage of a ward's informal residents without an improved water source, the percentage of a ward's informal residents without improved sanitation, distance to the nearest cholera treatment facility, population density, median asset index score in informal areas, and presence or absence of major roads. We found that cholera incidence was most closely associated with informal housing, population density, and the income level of informal residents. Using data available in this study, our model would suggest nearly a one percent increase in cholera incidence for every percentage point increase in informal residents, approximately a two percent increase in cholera incidence for every increase in population density of 1000 people per km^2^ in Dar es Salaam in 2006, and close to a fifty percent decrease in cholera incidence in wards where informal residents had minimally improved income levels, as measured by ownership of a radio or CD player on average, in comparison to wards where informal residents did not own any items about which they were asked. In this study, the range of access to improved sanitation and improved water sources was quite narrow at the ward level, limiting our ability to discern relationships between these variables and cholera incidence. Analysis at the individual household level for these variables would be of interest.

**Conclusions/Significance:**

Our results suggest that ongoing global urbanization coupled with urban poverty will be associated with increased risks for certain infectious diseases, such as cholera, underscoring the need for improved infrastructure and planning as the world's urban population continues to expand.

## Author Summary

In 2008, for the first time in human history, more than half of the world's population was living in urban areas, and this proportion is expected to increase. As a result of poor economic opportunities and an increasing shortage of affordable housing, much of the spatial growth in many of the world's fastest growing cities is a result of the expansion of informal settlements where residents live without security of tenure and with limited access to basic infrastructure. Although inadequate water and sanitation facilities, crowding, and other poor living conditions can have a significant impact on the spread of infectious diseases, analyses relating these diseases to ongoing global urbanization, especially at the neighborhood and household level in informal settlements, have been infrequent. To begin to address this deficiency, we analyzed urban environmental data and the burden of cholera in Dar es Salaam, Tanzania. We found that cholera incidence was most closely associated with informal housing, population density, and the income level of informal residents. Our analysis suggests that the current growth of many cities in developing countries and expansion of informal settlements will be associated with increased risks to human health, including cholera and other infectious diseases, and underscores the importance of urban planning, resource allocation, and infrastructure placement and management, as the rapidly progressive trend of global urbanization proceeds.

## Introduction

In 2008, for the first time in human history, more than half of the world's population was living in urban areas, and this proportion is expected to increase. Much of the future growth in urban areas is expected to take place in developing countries, with Africa and Asia predicted to have nearly seven out of every ten urban inhabitants in the world by 2030 [1]. Unfortunately, due in part to limited economic opportunities and an increasing shortage of affordable housing, the majority of urban growth in many of the developing world's fastest growing cities is a result of the expansion of informal settlements, often referred to as slums [2].

UN-HABITAT defines slums as urban areas where households lack one or more of the following conditions: access to an improved drinking water source, access to improved sanitation facilities, sufficient living area, durable housing in a non-hazardous location, and security of tenure [2]. As one might expect, these conditions can have severe consequences for human health and are of particular concern when considering their potential impact on the spread and burden of infectious diseases [3],[4]. For example, tuberculosis, influenza, meningitis, typhus, plague, typhoid and cholera are among many infectious diseases historically associated with conditions now common in urban informal settlements. In spite of this, few modern data are available assessing the current association of infectious diseases with ongoing global urbanization, especially at the neighborhood and household level in informal settlements [5],[6]. To begin to address this, we therefore analyzed urban environmental data and the burden of cholera in Dar es Salaam, Tanzania, a rapidly expanding African city.

**Figure 1:**
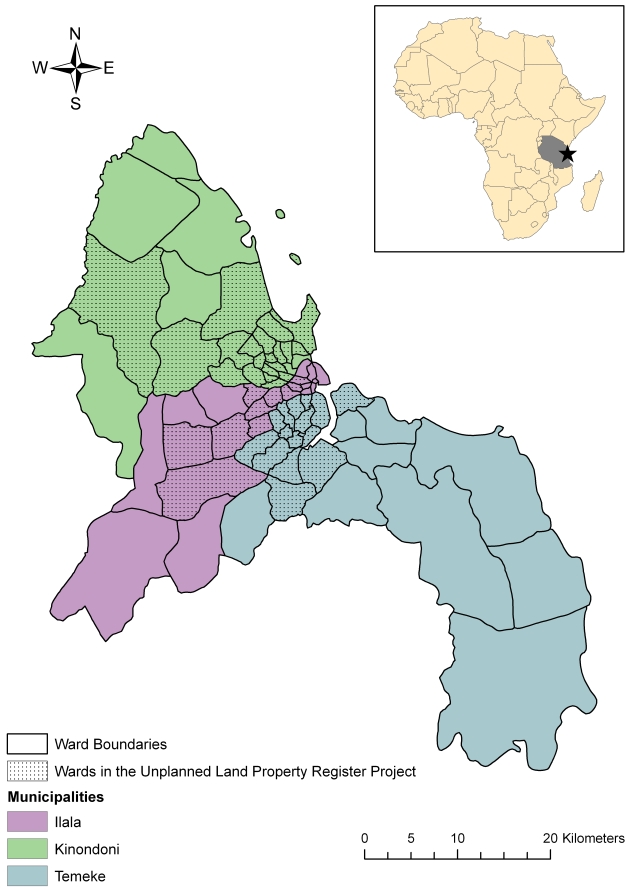
Municipal and ward boundaries in Dar es Salaam, Tanzania. A total of 45 wards were included in the Unplanned Land Property Register Project, and are indicated by stippling.

**Figure 2:**
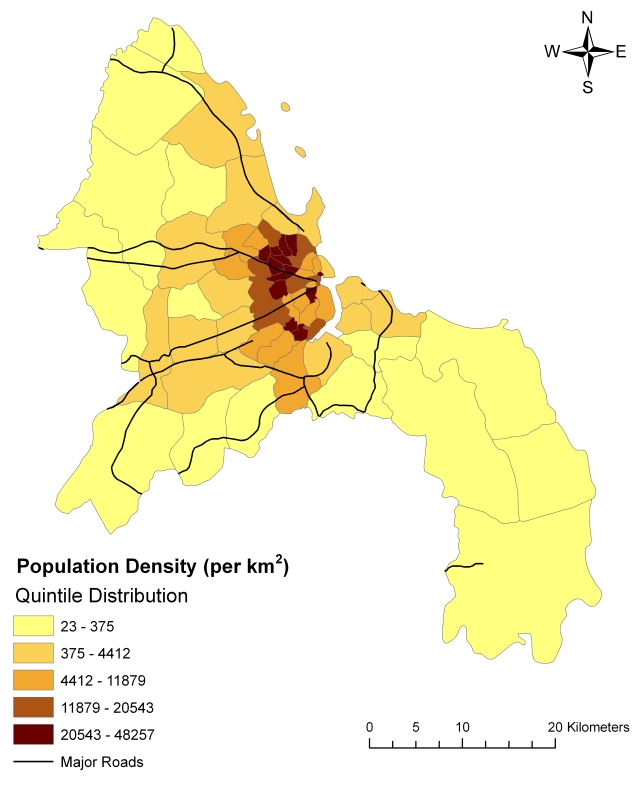
Population density by ward and major roads in Dar es Salaam, Tanzania, 2006. Ward populations in 2006 were projected by the National Bureau of Statistics using data from the 2002 Census.

Dar es Salaam, Tanzania's largest city, covers an area of approximately 1800 km^2^ situated alongside the Indian Ocean. The city is comprised of three municipalities – Kinondoni, Ilala, and Temeke – which are divided into 73 wards (Figure 1). The city grew from a population of 76,000 in 1950 to a population of 3.31 million in 2008 [7]. With a current estimated growth rate of 4.3% per year [8], Dar es Salaam contains 29% of the country's urban population, though this percentage is expected to nearly double by 2010 [7]. Most of the city's growth has occurred along the coastline and along four main arterial roads [9] (Figure 2). Despite increasing efforts on the part of municipal and city governments to address informal settlement expansion, the speed of the city's growth and the inability to invest adequately in housing and infrastructure have led to the growth of existing settlements and to the development of new ones. As a result, Dar es Salaam now has one of the highest proportions of informal-settlement households in East Africa, with 65% of households living in informal areas [7]. Investigating the relationship between cholera incidence and the size of and conditions in informal settlements may help to shed light on some of the health consequences of settlement expansion.

*Vibrio cholerae* is a water- and food-borne Gram-negative bacterium and the cause of cholera, a severe, dehydrating diarrhea in humans. *V. cholerae* is unique among the diarrheal pathogens because of its ability to cause global pandemics. The disease has a short incubation period of 18 hours to five days [10], and can thus spread rapidly through a population. Cholera may cause explosive outbreaks in crowded conditions, such as occurred in Goma in 1994 [11] and in Zimbabwe in 2009 [12]. In addition to causing epidemic disease, cholera is also endemic in many areas of the world, especially sub-Saharan Africa and South Asia. If untreated, mortality rates due to cholera can be as high as 50% [13], though nearly all deaths can be avoided if replacement fluids are promptly administered. Antibiotics may also be used to shorten the recovery period for severe cases [14]. Strains of *V. cholerae* can be differentiated serologically by the O side chain of the lipopolysaccharide (LPS) component of the outer membrane, and the strains that produce epidemic cholera belong to serogroup O1 or O139. *V. cholerae* O1 itself is classified into two biotypes, classical and El Tor, which differ clinically and biochemically. *V. cholerae* O1 biotype El Tor is responsible for the current seventh pandemic of cholera, and is the current cause of cholera in Dar es Salaam [15]. Although reporting may be incomplete, cholera is one of the few diseases that require reporting to the WHO under the International Health Regulations.

Though a cholera epidemic was first reported in East Africa in 1836, no cases were reported across Africa between 1870 and 1970 [16], when cholera returned to the continent as part of the seventh cholera pandemic, which started in Asia in 1961 [10]. Cholera cases associated with this most recent pandemic were reported for the first time in Tanzania in 1974, and have been reported each year since 1977. The first major outbreak in Tanzania occurred in 1992, although the largest country-wide epidemic occurred in 1997, with more than 40,000 cases reported. This epidemic was reported to have started in Dar es Salaam [15]. Of all regions in Tanzania, Dar es Salaam has had the most cholera cases since 2002. In an outbreak in 2006, Dar es Salaam was the most affected region, accounting for 63% of the total cases (14,297) and 40% of the total deaths (254) [15].

Cholera is most commonly caused by ingestion of water or food contaminated with fecal matter, and due to this mode of transmission, risk factors for cholera include lack of safe drinking water, poor sanitation, high population density, crowding, and lack of previous exposure, all of which are often common features in urban slum areas [17],[18]. In a number of countries, cholera incidence has been shown to be highest in highly urbanized areas [19]–[21]. We, therefore, chose to focus our analysis on the associations between cholera incidence and the nature of informal settlements in Dar es Salaam.

## Methods

### Data on cholera cases

In order to gain a sense of the general pattern in cholera cases in Dar es Salaam and to identify a time period that might be particularly useful for analysis, weekly suspected cholera case reports from 2006 through June 2008 were gathered from the City Health Officer and analyzed over time and by municipality. Suspected cases included all those patients assessed at one of the city's three cholera camps (described in detail below) whose symptoms were deemed to meet the WHO case definition for cholera in endemic areas: “a patient older than 5 years who develops severe dehydration from acute watery diarrhea (usually with vomiting); or any patient above the age of 2 years with acute watery diarrhea in areas where there is an outbreak of cholera” [13]. Suspected cases of cholera among children younger than 2 years of age were also included in analyses if confirmatory laboratory results were available. However, though ages of suspected cholera cases were available in Kinondoni, they were not available for the other two municipalities, and it was not possible to link suspected cases to confirmatory laboratory results for these areas. Based on data from Kinondoni, it was estimated that less than 2% of the sample was comprised of children under the age of 2 years.

Daily reports of suspected cases were collected from the District Medical Officer in each municipality, including information on the ward of residence, month and year. Where possible, information on the age and gender of suspected cases was also collected. Rectal swab results from those suspected cholera patients who were tested were also obtained from Amana Hospital in Ilala, from Temeke Hospital in Temeke, and from Mwananyamala Hospital in Kinondoni from 2006 through June 2008 in order to confirm the presence of *V. cholerae* O1 during months when suspected cases were being reported. Rectal swabs were collected at the discretion of the attending health provider as part of routine diagnostic assessments and were not collected for research purposes. Cholera incidence was calculated by ward for each year, using the available cholera count data and population projections for each ward based on the 2002 Census [22].

In comparison to the year 2006, in which 8,753 cases of cholera were reported in Dar es Salaam, only 395 cases were reported in 2007 and only 216 cases had been reported by the end of June in 2008. Due to the high volume of cholera cases in 2006, we focused our analysis on this year alone. Though the number of cases by municipality was available for all months in 2006, the location of cases by ward for the months of June through September was unable to be retrieved. However, 89.2% of the year's cases occurred in other months, including the months with the highest number of cases. Therefore, our analysis focused on these eight months. In addition, by using data from the Unplanned Land Property Register Project, analysis was limited to the 45 of the city's 73 wards included in the project; included areas were predicted to contain 84% of the city's population (Figures 1 and 2). Because the relationship between cholera incidence and predictors was nonlinear, cholera incidence was transformed into a natural logarithmic scale.

All potential cholera cases were sent to one of three cholera camps located in the city for further assessment and, if needed, treatment. These camps were located at Buguruni Health Center in Ilala, Mburahati Dispensary in Kinondoni, and at Temeke Hospital between Azimio and Tandika wards in Temeke. The geographic coordinates for these camps were obtained from the Tanzania Service Availability Mapping 2005–2006 [23]. In order to calculate population density and distance to the nearest cholera camp, all data were mapped in ArcMap 9.2 [24], utilizing a map file of wards in Dar es Salaam obtained from the International Livestock Research Institute [25]. Since we were not able to acquire individual addresses of suspected cholera cases, the distance to the closest cholera clinic was calculated, utilizing the center of the ward as the spatial reference. In addition, the area of each ward was calculated in square kilometers, and population density estimated based on the 2006 population estimates by ward. A shapefile of roads in Tanzania was acquired from the Food and Agriculture Organization's Multipurpose Africover Database [26], and the presence or absence of a major road in each ward was recorded as a categorical variable.

### Informal settlements data

Between 2004 and 2007, the Ministry of Lands and Human Settlements Development conducted a survey in informal settlements located in 45 wards as part of the Dar es Salaam Unplanned Land Property Register Project. The data were collected for administrative purposes. Data on 225,911 informal plots were gathered, mostly collected in 2005. Questionnaires were distributed to owners or close relatives, or, in the case of residences occupied by tenants only, tenants were questioned and the homeowner was notified in order to verify the information provided. Information was collected about plot use (residential, commercial, industrial, agricultural, other), the type and quantity of occupants, road access, source of water supply, type of sanitation, electricity, phone, building materials, length of time the occupants had lived in the area, ownership of household items and livestock, household income and type of employment, and garbage collection, among other characteristics.

Of 207,085 plots whose use was known, 98.2% contained residences. Of 203,289 residential plots, 186,631 were occupied at the time of the survey, 23.9% of which were in Ilala, 37.4% of which were in Kinondoni, and 38.8% of which were in Temeke. Only records for occupied residential plots were included for analysis. Among these records, 97.5% (181,896 records) contained complete information about a water source, while 61.4% (114,593 records) contained complete information about sanitation.

The number of informal residents in each ward was calculated by aggregating the number of occupants in each residential plot at the ward level. The percentage of informal residents in each ward was then calculated based on the 2006 population estimates. For three wards, estimates of the percent of the population that is informal were greater than 100. This is likely a result of rapid urban expansion that was underestimated by the population estimates. As a result, for these three wards, the maximum informal percentage obtained from other wards, which was 95.8%, was used. The percentage of informal residents in each ward was used as a proxy for the percent of residents in each area that would likely be living in areas with poorer environmental conditions.

Access to improved drinking water and access to improved sanitation were assessed based on guidelines from the WHO Joint Monitoring Program for Water Supply and Sanitation [27]. Thus, improved water sources included household connections, a neighbor's on-plot connection, and community pumps. Deep wells were also considered improved sources while shallow wells were not. The survey did not ask specifically about whether wells were protected, but due to the fact that shallow wells have a higher potential for contamination, particularly if they are uncovered, shallow wells were considered unimproved sources. Only sanitation facilities connected to the sewer system or to a septic tank were considered improved, as the type of pit latrine was not specified.

Due to the lack of a reliable measure of income among informal residents, an asset index was constructed in order to serve as a proxy for long-term economic status and to provide a more sensitive means of differentiating between poor households [28]. Following the example of Filmer and Pritchett [29], principle components analysis was used to generate weights for an index based on 22 asset indicators, including household ownership of consumer goods (air conditioner, bicycle, car, computer, DVD player, fan, furniture, iron, motorcycle, radio or CD player, refrigerator, sewing machine, stove, TV or video player, tools, truck) and household ownership of animals or livestock (goat or sheep, chicken, cow, pig, dog or cat, other animals). Only the first principal component was used, which explained 16.9% of the variance. The index was designed so that a score of zero would indicate possession of no assets, while the score would increase with the possession of increasing numbers of assets.

### Analytic approach

Data analysis was conducted using only de-identified data, which were analyzed at the ward level. As a way to assess whether environmental conditions in informal settlements had an effect on the city's pattern of cholera incidence, correlations were first calculated between the natural log of cholera incidence in 2006 and the percentage of a ward's residents who were informal, the percentage of a ward's informal residents without an improved water source, the percentage of a ward's informal residents without improved sanitation, distance to the nearest cholera camp, population density, median asset index score in informal areas, and presence or absence of a major road.

Including as predictors only those variables significantly correlated at the 95% confidence level with the natural log of cholera incidence, data on the number of cholera cases per ward in 2006 were used to estimate cholera incidence rate ratios associated with the percentage of a ward's residents who were informal, population density, the percentage of a ward's informal residents without improved sanitation, and median asset index score in informal areas. Since we sought to model the number of cholera cases, and considering that overdispersion was observed (i.e. the variance in the number of cholera cases exceeded the mean), a negative bionomial regression model was used for analysis, with the logarithm of a ward's population size included as an offset variable.

## Results

### Spatial patterns of cholera

**Figure 3:**
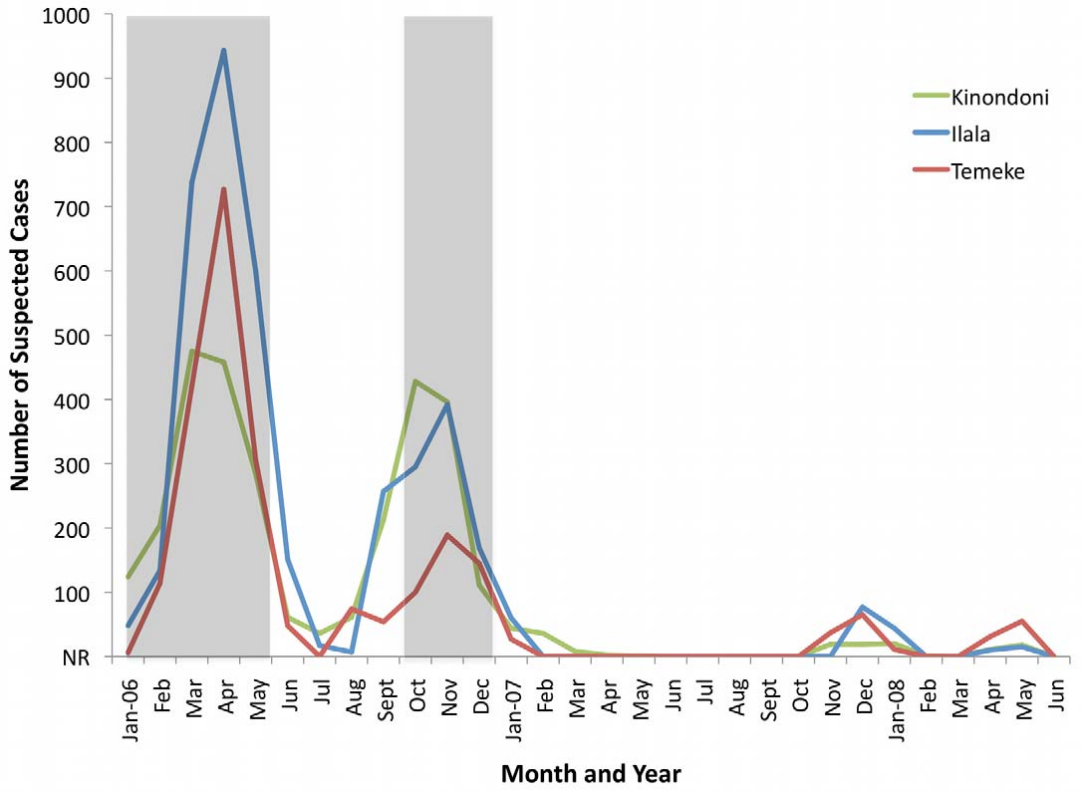
Number of suspected cholera cases by month, year, and municipality in Dar es Salaam, 2006–2008. “NR” indicates months for which no cholera cases were reported. The shaded areas indicate time periods for which data were available at the ward level.

**Figure 4:**
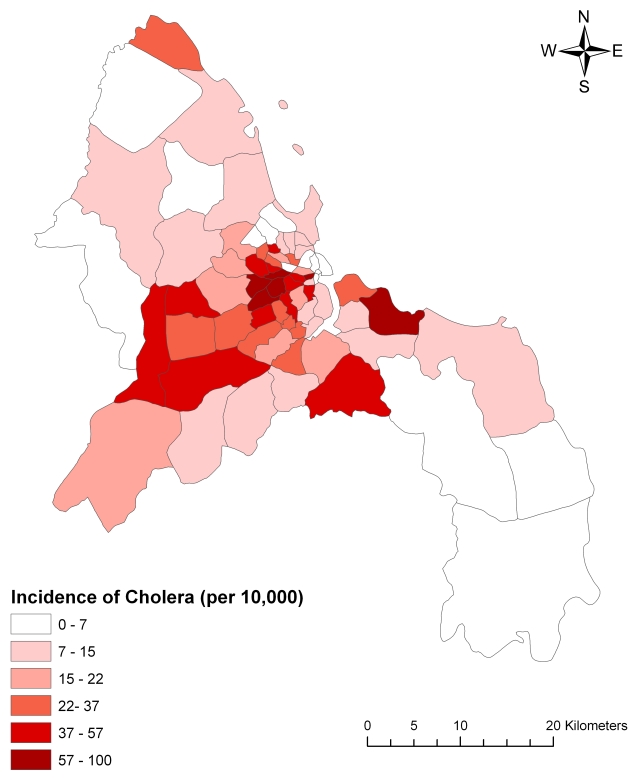
Cholera incidence in Dar es Salaam, 2006. Legend categories were determined by natural breaks.

In 2006, 8,753 cases of cholera were reported in Dar es Salaam, of which 42.8% were in Ilala, 32.5% in Kinondoni, and 24.7% in Temeke. The outbreak displayed two peaks – one in April and one in October (Figure 3). In any given month, *V. cholerae* O1 was identified by culture from rectal swabs taken from suspected cases in at least a third of those tested. The median incidence of cholera per 10,000 people in Dar es Salaam in 2006 was 15.8, with 25% percent of wards reporting an incidence of 7.8 cases or fewer per 10,000 people, and 25% of the wards reporting an incidence of 36.6 cases or higher per 10,000 people. The maximum incidence reported was 99.6, while one ward reported no cases (Figure 4).

Since the Unplanned Land Property Register Project was restricted to 45 wards, further analysis was restricted to this area. These wards contain the majority of urban areas in Dar es Salaam, and account for 84% of the city's population. For the 45 wards analyzed, the percentage of informal residents without access to improved drinking water ranged from 37.8% to 90.0%, with a mean of 71.8%. The percentage of informal residents lacking improved sanitation ranged from 71.7% to 97.3%, with a mean of 92.4%. The average percentage of informal residents per ward was 60.1, ranging from 5.4 to 95.8. The mean population density for these wards was 14,874 people per km^2^, ranging from a low of 334 people per km^2^ to a high of 48,257 people per km^2^. Distance to the nearest cholera clinic ranged from 0.4 km to 17.4 km, with a mean of 3.8 km. The median asset index score overall ranged from 0 in 26 wards to 0.7 in one ward. Among occupied residential households, 56.3% did not report ownership of any of the items about which they were asked (Table 1). Major roads crossed 27 of the 45 wards analyzed.

**Table 1:** Principal component analysis of assets reported by informal residents in 45 wards in Dar es Salaam, Tanzania.

Asset	Scoring Factor	% Ownership
Own Air Conditioner	0.156	1.0
Own Bicycle	0.203	4.6
Own Car	0.285	3.1
Own Computer	0.099	0.16
Own DVD Player	0.269	3.3
Own Fan	0.327	8.6
Own Furniture	0.016	0.41
Own Iron	0.075	0.72
Own Motorcycle	0.113	0.53
Own Radio/CD Player	0.324	38.7
Own Refrigerator	0.39	14.6
Own Sewing Machine	0.275	5.0
Own Stove	0.32	4.3
Own TV/Video Player	0.373	23.5
Own Tools	0.005	0.53
Own Truck	0.168	0.65
Own Dog/Cat	0.041	0.10
Own Goat/Sheep	0.078	0.46
Own Chicken	0.114	2.8
Own Cattle	0.111	0.93
Own Pig	0.074	0.51
Own Other Birds	0.05	0.46

Data collected as part of the Dar es Salaam Unplanned Land Property Register Project (2004–2007). The scoring factor is the “weight” assigned to each variable in the linear combination of the variables that constitute the first principal component. Of the sample, 56.3% of the sample did not report ownership of any of the items listed.

**Figure 5:**
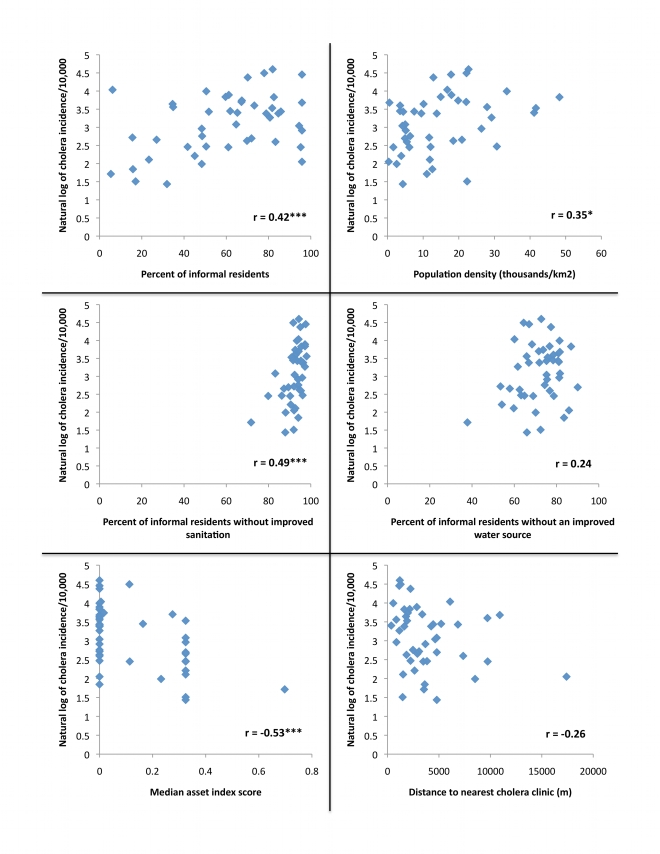
Scatter plots of the natural log of cholera incidence/10,000 population versus potential predictors among 45 wards in Dar es Salaam, Tanzania. As the presence of roads was a categorical predictor, it was not included here. Pearson's correlation coefficient between each variable and the natural log of cholera incidence/10,000 population is indicated. * p<0.05, ** p<0.01, *** p<0.001

Of all the variables considered (Figure 5), median asset index score was the most highly correlated with the natural log of cholera incidence (r = −0.53, p<0.001). The percentage of a ward's informal residents without access to improved sanitation, the percentage of informal residents in a ward and population density had moderate and positive effects on cholera incidence (r = 0.49, p<0.001, r = 0.42, p = 0.004 and r = 0.35, p = 0.02, respectively). In comparison, major road presence, distance to the nearest cholera clinic, and percentage of informal residents without access to improved water sources were not found to be significantly correlated with cholera incidence.

Results of the negative binomial regression model indicated that when all four predictors were included, the effects of percent of informal residents, population density, and median asset index score on cholera incidence were found to be significant, while the effect of percentage of informal residents without access to improved sanitation was not found to be significant (Table 2). Although additional data would strengthen any potential explanatory model, our analysis of available data and interpretation of coefficients of the negative binomial model suggested nearly a one percent increase in cholera incidence for every percentage point increase in informal residents, and approximately a two percent increase in cholera incidence for every increase in population density by 1,000 people per km^2^ in Dar es Salaam in 2006. At the same time, the model suggested nearly a fifty percent lower cholera incidence in a ward with a median asset index score of 0.32, which is equivalent to informal residents owning a radio or CD player, compared to a ward where the median asset index score was zero.

**Table 2:** Incidence rate ratios for the negative binomial model estimating cholera cases among 45 wards in Dar es Salaam, Tanzania in 2006, offset by the natural log of population.

Variables	Incidence Rate Ratio	95% Confidence Interval
Percent of Informal Residents	1.0075	1.0004–1.0147
Population Density (thousands/km^2^)	1.0228	1.0049–1.0411
Percent of Informal Residents without Improved Sanitation*	1.0085	0.9502–1.0704
Median Asset Score of Informal Residents	0.1301	0.02368–0.7144

*: Out of 186,631 occupied residential plots, 114,593 reported information on sanitation.

## Discussion

Through an analysis of the spatial patterns of cholera in Dar es Salaam, we found in this study that the extent of informal occupancy, population density, and income level had significant effects on cholera incidence in the city in 2006. Interestingly, in our analysis, we did not find an association between cholera incidence and access to improved water sources or improved sanitation at the ward level, despite the fact that cholera is transmitted fecal-orally. This may be due to the fact that, as shown in Figure 5, the range of access to improved sanitation and improved water sources was quite narrow at the ward level in Dar es Salaam in 2006, limiting our ability to discern relationships between these variables and cholera incidence. Analysis at the individual household level for these variables would be of interest. Poverty has, however, been closely linked to inadequate water and sanitation in the past [30], and our detection of an association between median asset index score and cholera incidence may reflect in part the level of access to improved water and sanitation in this study. Underscoring the relationship between cholera and extreme poverty, our study suggests that relatively minor increases in income can correlate with a marked decrease in the risk of cholera.

Our observed association of cholera incidence with population density and the extent of informal settlement may also reflect in part the increased risk of acquiring *V. cholerae* recently passed by another human in a densely populated urban slum area, compared to the risk of acquiring *V. cholerae* from ecological reservoirs not recently contaminated by humans. *V. cholerae* can exist in environmental water sources independent from humans, especially in association with zooplankton and phytoplankton, and these organisms may serve as an important reservoir for infection [10],[31]. However, the passage of *V. cholerae* through a human intestine leads to a transient hyperinfectious bacterial state that can persist for up to 24 hours in environmental reservoirs, and such hyperinfectiousness may contribute significantly to human-to-human transmission [32]–[36].

Individuals in densely populated urban slums characterized by poor sanitary conditions may be at particular risk of ingesting such hyperinfectious *V. cholerae*, of therefore becoming involved in explosive outbreaks and epidemics, and of contributing to ongoing human-to-human transmission. Interestingly, recent data suggest that *V. cholerae* loses a significant degree of its hyperinfectiousness within hours of passage from a human intestine, suggesting that even in densely populated urban slums, relatively minor modifications in water usage patterns (such as securely storing water for perhaps as short as a day prior to ingestion to allow *V. cholerae* to revert to its non-hyperinfectious state) could possibly have significant impact on the burden of cholera in a slum area [37].

By suggesting that variations in the economic and physical conditions between and within informal settlements may translate into variations in disease patterns, this study validates previous findings that statistical analysis at the city level may mask important intra-urban differences. For example, infant and child mortality rates among Nairobi's slum populations were found to be three to four times higher than the city's average, and higher even than the average for rural areas in Kenya [38]. In Accra, Ghana, the relative risk of infectious and parasitic diseases was also found to be twice as high in areas of the city with the worst social and physical environmental conditions compared to the burden in the best areas [39]. Furthermore, this study adds to the growing body of research demonstrating that even within and between informal settlement areas, relatively small variations in social and physical conditions can result in markedly different rates of infection [18],[40],[41].

This pattern resembles the conditions resulting from rapid growth of European cities in the eighteenth and nineteenth centuries, which often led to a poorer health environment in urban areas [42]. However, the rate at which urban growth has been and is occurring in developing countries is unprecedented, and the resources available to address the myriad issues coupled with this growth are often extremely limited [4],[43]. Although urban-associated infectious disease burden will predominantly be borne by the poorest in many of the world's fastest growing cities, the increased movement of people within and between population centers and countries will also facilitate the spread of these diseases between poor urban centers and other areas and populations [3]. Again, using cholera as an example, between 2006 and 2007, more than 82,000 cases of cholera and 3,000 deaths were reported in Angola, the worst outbreak ever reported in that country [44]. The epidemic was reported to have started in one of the poorest and most overcrowded informal settlements in the capital city of Luanda and, over the course of the epidemic, spread to 16 of the country's 18 provinces [45].

Our study has a number of limitations. We analyzed cholera data collected during an epidemic, and patterns of endemic disease and factors involved in its transmission during endemic periods might be different than those we assessed. However, our use of data during the selected period allowed us to analyze a sufficient cholera case burden in the context of our measured environmental parameters. We used extant data sets reporting cholera burden that themselves were largely based on a syndromic classification system using WHO criteria. It is possible that cases classified as cholera where not caused by *V. cholerae* infection, and that other actual cases of cholera were not captured in the municipal reporting system; however, all cases included in this analysis met WHO criteria, microbiologic data were highly supportive when performed, and reporting criteria did not change during the period of data collection. In addition, though the negative binomial model used in this analysis accounted for high variability in the number of cholera cases, it did not account for any spatial structure that might have been present in the data. Given that our model tended to underestimate the number of cholera cases in the center of the city while overestimating the number of cases farther from the center, a spatial model might have provided additional insight, but was not able to be used given the small number of wards for which data were available. By using an extant data set, we were also limited by incomplete survey information; however, we felt that the ability to analyze an urban environmental survey undertaken immediately prior to an urban cholera epidemic provided a unique opportunity for analysis and, at least in part, mitigated this limitation.

Similarly, while we may have obtained a more robust understanding of the risk of cholera had we analyzed data at the household or individual level (as opposed to the ward level), such data were not available. Such an analysis may have shown that access to specific water sources or certain types of sanitary conditions or behaviors may affect the risk for cholera within a household. For instance, in a randomized control trial in low-income squatter settlements with poor water and sanitation in urban Pakistan, children in households encouraged to wash their hands with soap had a 53% lower incidence of diarrhea than in control households [46].

It should also be mentioned that we made the assumption in this analysis that people who became ill with cholera did so due to conditions in their area of residence, while it may also be the case that where people work and the types of activities they engage in away from home may have an effect on their risk of diarrheal illness. For example, consumption of food prepared away from the home is increasing in many developing countries, and was found in Nairobi to be associated with the distance one worked from the home. Preparation of these foods often involves questionable hygienic practices, which may increase the risk of diarrheal disease for those who consume them [47]. However, during an outbreak such as the one in 2006 in Dar es Salaam, the home environment may well play an important role in the spread of the disease.

In order to determine whether the relationship between environmental conditions and cholera in Dar es Salaam is unique or indicative of a more general relationship between environmental conditions and the risk for infectious disease, it would also be useful to compare the pattern of cholera incidence with patterns of other diseases across the city to see whether the nature and spatial pattern of risks are similar. For instance, one might predict that the risk for other enteric diseases such as shigellosis might similarly correlate with population density and sanitation, while tuberculosis and influenza might correlate with crowding, and vector-borne diseases such as arboviral infections and malaria might correlate more with water sources, vector biting habits, and human behavior.

Equally important, as the municipalities have taken important steps in recent years to address conditions in informal areas, evaluating the impact of these efforts on the incidence of disease should be a crucial part of determining their effectiveness. For instance, in at least one example of informal settlement upgrading, improvements in health have already been noted. Specifically, the upgrading of an informal settlement in Hanna Nassif ward in Dar es Salaam from 1994 to 1998, which focused on the installation of water vending kiosks, the management of solid waste collectors, and construction of improved access roads and storm water drainage channels, led to a 39% reported decline in waterborne diseases from the start to the finish of the project [48].

In every developing region of the world excluding North Africa, slum growth is expected to closely match the rate of city growth [2]. In sub-Saharan Africa, 59 cities are expected to exceed one million inhabitants by 2015, with 43% of urban inhabitants living below the poverty line [7]. Our analysis suggests that the ongoing growth of many of the world's cities and expansion of informal settlements will be associated with increased risks to human health, including cholera and possibly other infectious diseases, and underscores the importance of urban planning, resource allocation, and infrastructure placement and management as the rapidly progressive trend of global urbanization proceeds.
